# Effect of Chronic Training on Heart Rate Variability, Salivary IgA and Salivary Alpha-Amylase in Elite Swimmers with a Disability

**DOI:** 10.1371/journal.pone.0127749

**Published:** 2015-06-04

**Authors:** Rohan Edmonds, Brendan Burkett, Anthony Leicht, Mark McKean

**Affiliations:** 1 School of Health and Sport Sciences, University of the Sunshine Coast, Sippy Downs, Queensland, Australia; 2 College of Healthcare Sciences, James Cook University, Douglas, Queensland, Australia; University of Rome Foro Italico, ITALY

## Abstract

The purpose of this study was to a) determine the heart rate variability (HRV) and saliva markers of immunity (salivary immunoglobulin A; sIgA) and stress (salivary alpha-amylase; sAA) responses to chronic training in elite swimmers with a disability; and b) identify the relationships between HRV, sIgA, sAA and training volume. Eight members of a high performance Paralympic swimming program were monitored for their weekly resting HRV, sIgA and sAA levels in the 14 weeks leading up to a major international competition. The 14 week training program included aerobic, anaerobic, power and speed, and taper training phases, while also incorporating two swimming step tests and two swimming competitions. Specific time (root mean square of the successive differences; RMSSD) and frequency (high frequency normalized units [HFnu]) domain measures, along with non-linear indices (standard deviation of instantaneous RR variability; SD1 and short term fractal scaling exponent; α1) of HRV were used for all analyses with effects examined using magnitude-based inferences. Relationships between HRV and saliva markers were identified by Spearman rank rho (ρ) correlation coefficients. Compared with week 1, SD1 was very likely lower (96/4/0, ES = -2.21), while sAA was very likely elevated (100/0/0, ES = 2.32) at the beginning of week 7 for all athletes. The training program did not alter HRV or saliva whereas competition did. There were also no apparent differences observed for HRV, sIgA and sAA between each of the training phases during the 14 week swimming program. Correlations were observed between sAA and SD1 (ρ = -0.212, p<0.05), along with sAA and mean HR (ρ = 0.309, p<0.05). These results show that high level national competition influences depresses HRV (SD1) and increases saliva biomarkers of stress (sAA). It appears that a well-managed and periodised swimming program can maintain these indices within normal baseline levels. The study also highlighted the parasympathetic nervous system influence on sAA.

## Introduction

Coaches and sport scientists are constantly striving to optimise athlete performance. Understanding how individual athletes respond to the weekly rigours of training and varying training intensities is imperative for coaches. The ability to appropriately monitor and adjust training programs helps in avoiding maladaptation and ultimately improves training and performance [1]. One such tool that has shown promise in monitoring training load is heart rate variability (HRV), a non-invasive means to assess cardiac autonomic activity in athletic populations [2,3].

Recently, HRV has been used in elite athlete populations to evaluate training adaption [1,4] with HRV increases and decreases reflecting changes in athlete fitness and freshness respectively [4]. Further, HRV monitoring throughout seasonal training has assisted with identification of individual training adaptations and/or early signs of maladaptation [5]. It has been suggested that long-term documentation of HRV is essential to understand an athlete’s unique HRV profile [4] with day-to-day variations in HRV a useful indicator of training maladaptation that may lead to non-functional overreaching (NFOR) [6]. However, similar longitudinal documentation of HRV in elite athletes with a disability has shown minimal day-to-day variations in HRV with the weekly average for HRV indices similar to the daily values over a 17 week monitoring period [7].

While HRV has shown potential as a non-invasive means to identify training adaptations [4], it does require extensive data analysis that limits on-the-spot feedback. With recent updates in technology, point of care collection and analysis of other physiological responses such as salivary biomarkers is now a possibility for real-time feedback to athletes and coaches. Salivary measures such as salivary immunoglobulin A (sIgA) have shown great promise to assist coaches and athletes as sIgA represents the first line of defence in mucosal immunity [8]. Several studies of athlete populations have shown that engaging in long-term strenuous training leads to a reduction of sIgA levels [9,10] with monitoring sIgA crucial for identifying the risk of infection in elite athletes [11]. Typically, sIgA levels in healthy elite able-bodied swimmers range between 43 μg/ml and 136 μg/ml at rest with illness causing a drop to between 22 μg/ml and 92 μg/ml [12,13,14]. While sIgA provides a quick and effective marker of athlete immune function [11], it may not be sensitive enough to identify training induced stress [15]. Therefore, other measures may provide a more sensitive indication of athlete training that remain to be clarified.

In the same way that point of care technology is able to provide on the spot feedback of sIgA, real-time analysis can now be utilized to measure salivary alpha amylase (sAA), a biomarker of stress and sympathetic nervous system activity [16]. First reported by Gilman and colleagues [17], sAA responses have been documented following various forms of acute exercise/events [18,19] with near immediate increases evident following cycling [20], running [21], rowing [22], marathon [18] and a cross country race [23]. Monitoring sAA responses has been suggested as a superior marker of exercise induced stress compared to cortisol as it reflects activity of the autonomic nervous system, a key regulatory system of the body [24]. Subsequently, the stress induced by acute exercise may be accurately assessed by sAA to assist with optimal prescription of training and recovery cycles in athletes [11]. In healthy, physically active adults, sAA generally ranges between 50 U/ml and 175 U/ml at rest with stress inducing a two-fold increase to between 110 U/ml and 350U/ml [25,26]. With an immediate recovery response, sAA generally returns to baseline within 30 to 60 minutes following the applied stressor [22]. To date there has been limited investigation of sAA responses to chronic training and less known about sAA responses to variations in training loads and intensities. Understanding the chronic responses of sAA to various training intensities will allow coaches to better prescribe periods of lighter training or recovery, with the aim to avoid training maladaptation and ultimately improve athletic performance. With Paralympic sport encompassing various athletes with contrasting disabilities, ranging from amputee athletes to athletes with cerebral palsy or neuromuscular impairments who often compete against each other, it is imperative that coaches are able to understand their athletes responses to training and competition.

The main aims of this study were to: a) determine the HRV, sIgA and sAA response to training in elite Paralympic swimmers; and b) indentify the relationship between HRV, saliva markers of immune function and training load in elite Paralympians.

## Methods

### Participants

Research ethics approval was granted by the Human Research Ethics Committee (HREC) of the University of the Sunshine Coast, HREC approval number: S/12/393. Written informed consent from all athletes was attained prior to participation. All athletes (n = 8) recruited for this study were members of an elite Paralympic swimming squad training an average of 25 hours per week. A normal week of training comprised of nine pool sessions of approximately two and a half hours duration each (22.5 hours weekly) and two strength sessions of 75 minutes duration (2.5 hours weekly). All athletes competed in sprint distance events (<200m) and had competed at the national level (including national and international competition) in the previous 12 months. All athletes followed periodised swimming programs that were individually prescribed by the head swimming coach. Descriptive statistics and impairment type for each athlete are presented in Table 1 with International Paralympic Committee (IPC) swimming classification code.

**Table 1:** Athlete Characteristics.

	Gender	Age (yrs)	Athlete (Classification)^a^	Disability	National Experience (yrs)^b^
**Athlete 1**	M	23	S8	Amputee	6
**Athlete 2**	M	27	S10	Neuromuscular	11
**Athlete 3**	M	19	S13	Vision	1
**Athlete 4**	M	29	SM10	Amputee	11
**Athlete 5**	M	17	S13	Vision	1
**Athlete 6**	M	16	S9	Amputee	1
**Athlete 7**	F	19	S14	Intellectual	2
**Athlete 8**	F	15	S8	Cerebral Palsy	1

^a^IPC Classification code

^b^Years competing as part of the national Paralympic swimming squad.

Each athlete was monitored over 14 weeks to examine the long-term effect of training on heart rate (HR) and saliva markers in the lead up to the 2014 Commonwealth Games (Glasgow, Scotland) and Pan Pacific Para-swimming championships (Pasadena, California). During the study period, athletes competed at two swimming grand prix meets (weeks 6 and 9) and completed two incremental swimming step tests (10x100m efforts) at weeks 3 and 11 (Table 2). Resting HR was recorded and saliva samples obtained on the first Monday morning of each training week prior to training for each of the 14 weeks.

**Table 2:** Average weekly kilometres completed during the 14 week monitoring period.

Training Phase	Week														
	1	2	3	4	5	6	7	8	9	10	11	12	13	14	15
**Aerobic Maintenance**	28	**-**	**-**	**-**	**-**	**-**	**-**	**-**	**-**	**-**	**-**	**-**	**-**	**-**	**-**
**Aerobic Capacity**	**-**	34	43	47	48	**-**	**-**	**-**	**-**	**-**	**-**	**-**	**-**	**-**	**-**
**Anaerobic Capacity**	**-**	**-**	**-**	**-**	**-**	45	48	48	45	47	**-**	**-**	**-**	**-**	**-**
**Power & Speed**	**-**	**-**	**-**	**-**	**-**	**-**	**-**	**-**	**-**	**-**	46	35	**-**	**-**	**-**
**Taper**	**-**	**-**	**-**	**-**	**-**	**-**	**-**	**-**	**-**	**-**	**-**	**-**	28	29	**-**
**Competition**						**X**			**X**				**X**		**X**
**Swimming Test**			**X**								**X**				

X *indicates competition or swimming test*

The swimming specific training phases incorporated in the current study included periods of aerobic maintenance, aerobic capacity, anaerobic capacity, power and speed, and taper. The aerobic maintenance phase was a recovery based training period with training HR aimed at being below 140 beats per minute (bpm). The aerobic capacity training phase consisted of lower intensity training sessions, with shorter rest periods and a training HR aimed at between 140–170 bpm, while the anaerobic capacity training phase focussed on higher intensity training sessions with extended rest periods and a training HR aimed at 170–180 bpm. The power and speed training phase incorporated periods of higher intensity training sets with moderate recovery periods and kilometres completed.

### Procedures

At the start of each week, overnight HR recordings (21:00–05:00) were acquired via a polar RS800CX (Polar Electro Oy, Finland, 1000Hz) with all HR data analysed using Kubios HRV software (v2.1, University of Kuopio, Finland). To ensure a consistent analysis for all athletes, HRV was calculated from the most stable (visually inspected for HR change of less than 10 beats) two hour time period between 23:00–04:00 during night time sleep. Along with mean HR, analysis of HRV included time-domain (root mean square of the successive differences; RMSSD), frequency domain (high frequency normalized units [HFnu]) and non-linear indices (standard deviation of instantaneous RR variability; SD1 and short term fractal scaling exponent; α1) as previously described [2]. Prior to the commencement of training, saliva samples were obtained from each athlete using an IPRO oral fluid collector (OFC; IPRO Interactive, Oxfordshire, UK). The OFC contains a volume adequacy indicator with a colour change evident (white to dark blue) once 0.5ml of saliva has been collected. Immediately after collection, each saliva sample was assessed for sIgA and sAA concentrations using an IPRO lateral flow device (IPRO Interactive, Oxfordshire, UK) point of care system as previously described [27]. This method of saliva analysis has previously been validated against ELISA analysis (R^2^ = 0.78) [28].

Daily training session information (main training set and distance completed) obtained from the head coach was documented and used to calculate daily and weekly training volume. Training was conducted in phases with specific focuses as part of each athlete’s periodised training program in consultation with the head swimming coach (Table 2).

### Statistical Analysis

The Statistical Package for Social Sciences (SPSS) software (v21, SPSS INC., Chicago, WI, USA) was used to analyse data. Data were expressed as mean variance from the baseline (90% Confidence Interval) with an alpha level established at p<0.05 for all analyses. All data were log-transformed prior to analysis to minimise bias arising from non-uniformity in error [29] as HRV has been previously reported to be not normally distributed [4,30]. The practical significance of changes in the data was assessed using magnitude-based inferences (MBI) [31].

The magnitude of the weekly change from baseline was assessed using standardised differences in means (i.e. Effect Size, ES) with threshold values established for ES as *small* (0.2), *moderate* (0.6), *large* (1.2), and *very large* (2.0) [30]. Magnitude based inferences were undertaken using the smallest worthwhile change or coefficient of variation calculated as 0.2 of the between-subjects standard deviation. Age was considered a potential influencing factor, and was taken into account within the MBI analysis. The chances of significant positive, trivial, or negative changes were assessed qualitatively as follows: *almost certainly not* (<0.5%); *very unlikely* (0.5%-5%); *unlikely* (5%-25%); *possibly* (25%-75%); *likely* (75%-95%); *very likely* (95%-99.5%); *almost certainly* (99.5%). The true difference was deemed unclear if the chances of having positive and negative changes were both greater than 5%.

Relationships between HRV, saliva variables and training volume were examined via Spearman’s rank rho (ρ) order correlations. Spearman’s rank rho (ρ) values closer to 1 indicated a strong agreement between variables, while a ρ value closer to -1 implied a strong negative agreement. A ρ value near zero implied there was no correlation between variables.

## Results

**Fig 1:**
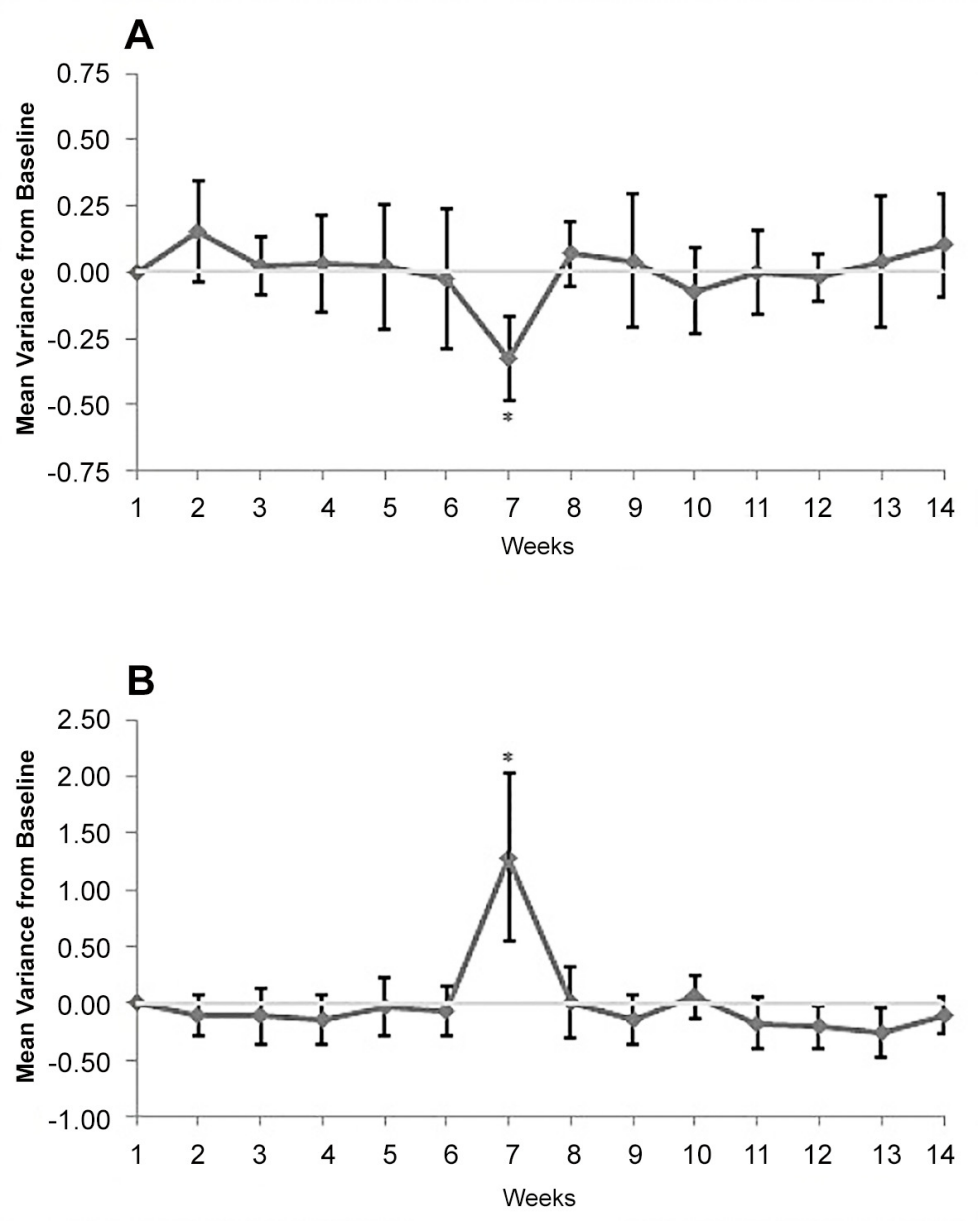
Percentage variance from the baseline (with 95% confidence interval) over the 14 week monitoring period for A) weekly heart rate variability, assessed by the standard deviation of short axis of the Poincare plot (SD1), and B) salivary alpha-amylase (sAA). * *indicates very likely lower (A) or very likely higher (B) than baseline*.

Changes in mean HR, RMSSD, α1 and HF(nu) during the monitoring period were trivial or unclear, and of small ES (Table 3). In contrast, SD1 was very likely lower (96/4/0, ES = -2.21) at the beginning of week 7 for athletes compared to baseline (Fig 1A).

**Table 3:** Mean values, variance from baseline, Cohen’s effect size (ES) and qualitative inferences for heart rate variability and saliva measures during the 14 week monitoring period.

		Week												
	Baseline	2	3	4	5	6	7	8	9	10	11	12	13	14
**Mean HR (bpm)**	**57.5**	**57.0**	**57.2**	**55.8**	**57.8**	**56.2**	**58.5**	**57.2**	**57.1**	**55.2**	**56.8**	**59.2**	**58.4**	**57.2**
%Δ		-0.7	-0.7	-3.1	0.4	-2.2	1.3	-0.6	-0.8	-3.4	-1.4	3.4	1.9	-0.3
(± 90% CI)		(± 4.9)	(± 3.2)	(± 5.3)	(± 6.3)	(± 4.9)	(± 6.5)	(± 3.8)	(± 8.6)	(± 2.9)	(± 4.5)	(± 8.0)	(± 8.7)	(± 9.5)
ES		-0.04	-0.04	-0.17	0.02	-0.12	0.07	-0.03	-0.04	-0.21	-0.08	0.19	0.10	-0.02
QI		**Unclear**	**Trivial**	**Trivial**	**Unclear**	**Trivial**	**Unclear**	**Trivial**	**Unclear**	**Trivial**	**Trivial**	**Unclear**	**Unclear**	**Unclear**
**RMSSD (ms)**	**73.8**	**78.6**	**77.4**	**76.9**	**73.7**	**70.9**	**66.6**	**72.6**	**76.4**	**69.7**	**80.6**	**65.9**	**71.5**	**72.0**
%Δ		7.4	3.1	2.9	-1.4	-4.4	-13.8	-2.4	3.1	-8.8	10.0	-12.9	-3.7	1.0
(± 90% CI)		(± 8.9)	(± 6.6)	(± 11.5)	(± 15.7)	(± 16.7)	(± 19.0)	(± 10.9)	(± 15.8)	(± 10.9)	(± 8.6)	(± 10.4)	(± 13.9)	(± 14.2)
ES		0.15	0.06	0.06	-0.03	-0.09	-0.31	-0.05	0.06	-0.19	0.20	-0.29	-0.08	0.02
QI		**Trivial**	**Trivial**	**Trivial**	**Unclear**	**Unclear**	**Trivial**	**Trivial**	**Unclear**	**Trivial**	**Trivial**	**Trivial**	**Unclear**	**Unclear**
**α1**	**0.9**	**0.9**	**1.0**	**1.0**	**0.9**	**0.9**	**0.9**	**1.0**	**0.9**	**0.9**	**0.9**	**1.0**	**0.9**	**1.0**
%Δ		3.1	7.3	5.7	5.5	4.3	-0.3	10.2	-0.5	2.7	2.5	10.5	4.4	7.9
(± 90% CI)		(± 6.0)	(± 4.7)	(± 11.9)	(± 5.3)	(± 9.1)	(± 12.8)	(± 8.7)	(± 10.8)	(± 9.1)	(± 9.2)	(± 9.7)	(± 9.3)	(± 8.7)
ES		0.11	0.25	0.20	0.19	0.15	-0.01	0.35	-0.02	0.10	0.09	0.36	0.15	0.27
QI		**Trivial**	**Trivial**	**Unclear**	**Trivial**	**Trivial**	**Unclear**	**Trivial**	**Unclear**	**Unclear**	**Unclear**	**Trivial**	**Trivial**	**Trivial**
**HF(nu)**	**47.7**	**45.7**	**44.0**	**42.5**	**44.5**	**45.0**	**46.4**	**42.2**	**48.4**	**47.2**	**46.1**	**42.3**	**46.1**	**43.3**
%Δ		-4.4	-8.6	-14.4	-8.4	-7.2	-6.0	-12.3	3.7	-1.7	-4.5	-14.9	-2.8	-9.8
(± 90% CI)		(± 6.7)	(± 6.6)	(± 11.7)	(± 5.0)	(± 11.5)	(± 11.5)	(± 7.6)	(± 16.8)	(± 10.8)	(± 7.7)	(± 9.7)	(± 11.8)	(± 8.0)
ES		-0.09	-0.18	-0.31	-0.18	-0.15	-0.12	-0.26	0.07	-0.04	-0.09	-0.32	-0.06	-0.21
QI		**Trivial**	**Trivial**	**Trivial**	**Trivial**	**Trivial**	**Trivial**	**Trivial**	**Unclear**	**Unclear**	**Trivial**	**Trivial**	**Unclear**	**Trivial**
**sIgA (μg/ml)**	**43.4**	**53.6**	**46.4**	**51.8**	**40.1**	**50.4**	**51.3**	**43.7**	**54.0**	**52.1**	**58.2**	**54.1**	**52.9**	**55.7**
%Δ		21.6	3.6	17.5	-14.5	5.6	13.3	-6.1	16.0	12.9	29.4	22.7	19.9	28.0
(± 90% CI)		(± 17.6)	(± 20.9)	(± 19.1)	(± 27.5)	(± 29.6)	(± 25.5)	(± 23.6)	(± 28.6)	(± 25.9)	(± 24.2)	(± 26.2)	(± 24.4)	(± 22.3)
ES		0.74	0.13	0.61	-0.60	0.21	0.48	-0.24	0.56	0.46	0.98	0.78	0.69	0.94
QI		**Trivial**	**Unclear**	**Trivial**	**Unclear**	**Unclear**	**Unclear**	**Unclear**	**Unclear**	**Unclear**	**Trivial**	**Trivial**	**Trivial**	**Trivial**

Root mean square of the successive differences—RMSSD; short term fractal scaling exponent— α1; high frequency normalised units—HF(nu); salivary immunoglobulin A—sIgA

%Δ—Percentage variance from baseline; CI—Confidence Interval; ES—Effect Size; QI—Qualitative Inference

In a similar way to most of the HRV measures, changes for sIgA were trivial or unclear over the 14 week monitoring period (Table 3). However, sAA was very likely (100/0/0, ES = 2.32) elevated at the beginning of week 7 for athletes compared to baseline (Fig 1B).

**Fig 2:**
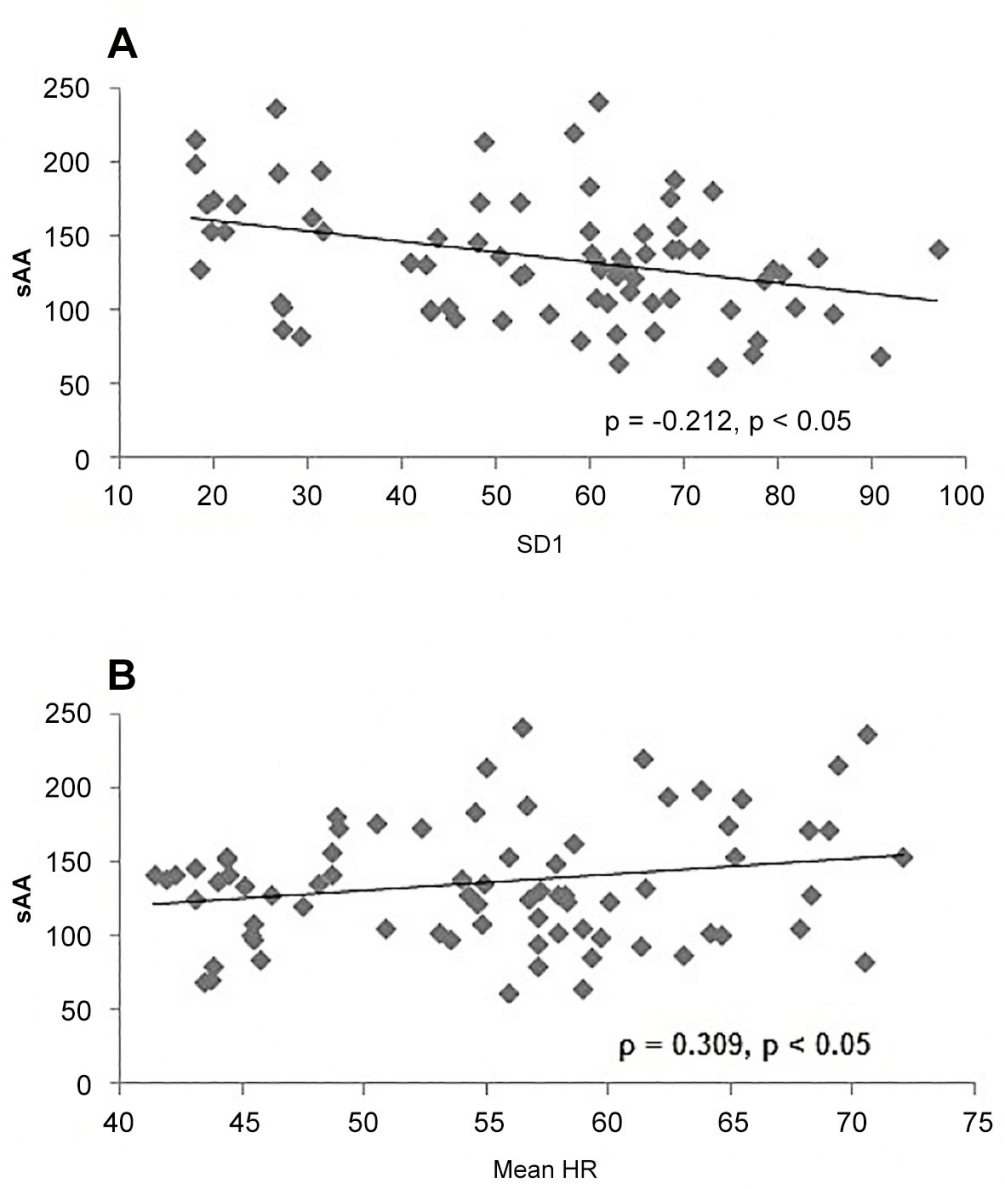
Relationship between salivary alpha-amylase (sAA) and A) weekly heart rate variability, assessed by the standard deviation of short axis of the Poincare plot (SD1), and B) weekly heart rate (HR). *ρ—Spearman’s rank rho correlation coefficient*.

Significant negative correlations were identified over the monitoring period between weekly mean HR and RMSSD (ρ = -0.738, p<0.05) and SD1 (ρ = -0.720, p<0.05). A similar negative correlation was observed between weekly sAA and SD1 (Fig 2A). In contrast, a significant positive correlation was identified between weekly sAA and mean HR (Fig 2B). Correlational analysis demonstrated no significant relationship between HRV indexes and years of experiences (p>0.05).

## Discussion

The current research was the first of its kind to investigate both weekly HRV and saliva (sIgA and sAA) responses to chronic training in a unique athletic population. The present study established that high level national competition decreases HRV (SD1) and increases saliva biomarkers of stress (sAA). Secondly, the periodised swimming program used in this study did not elicit a statistically significant shift in cardiac autonomic activity or immune function in elite swimmers with a disability (outside of competition). Lastly, the current study was the first to document a significant negative relationship between HRV (SD1) and sAA. Prior to the start of the 14 week monitoring period all swimmers had a 2 week recovery period where they did not train. This recovery period likely eliminated any potential for pre-agonistic stress before the baseline recording. In addition, the athletes were relatively experienced and accustomed to the rigors of weekly training and competition cycles, potentially limiting stress responses leading into the monitoring period.

The current study analysed weekly HRV in line with previous research [7,32]. Edmonds and colleagues [7] observed no significant difference between the weekly average and daily measures for HRV (RMSSD, SD1, α1 and HFnu) in elite Paralympic medallist swimmers during a long-term (17 week) monitoring period leading into the London 2012 Paralympic Games. From a coaches perspective, weekly analysis of HRV is more practical and appears to still be able to provide quality data to assist coaches in monitoring long term training response [7].

Interestingly, the only shift in both HRV (SD1) and saliva biomarkers (sAA) during the 14 week monitoring period was evident following the first competition weekend. As a measure of predominately parasympathetic activity, decreases and increases in SD1 have previously been used to indicate fatigue [33] and training induced changes [34] of HRV in an athlete populations. In line with previous studies [2,35] athletes exhibited a sustained depression in HRV, accompanied by an elevation in sAA after a three day national grand prix competition inclusive of 5 events for each swimmer. Comparable HRV changes have been reported with elite youth rugby league players also displaying an altered cardiac autonomic regulation with HF (nu) significantly reduced up to 48 hours after a competitive rugby league match [2]. Increased levels of cortisol following a rugby league match [35] provides further support that high level competition impacts on cardiac autonomic and metabolic systems for at least two days, which may impact on subsequent training and performance. While the time difference between the last exercise bout and HRV recording was smaller during the competition weekend (18 hours compared to 40 hours) this was not likely to have influenced the results of the current study. Previous research has shown HRV to have been reduced up to 48 hours after competition [2], suggesting that if the recording had occurred at 40 hours post competition (as per normal training weeks), the HRV indices would likely have still been reduced.

Understanding the sustained impact of competition on HRV and saliva markers of stress will allow coaches to integrate various recovery methods to alleviate the accumulated stress following competition [36]. As part of the athletes’ normal recovery routines, the current study incorporated a range of activities such as ice baths, massage therapy and lighter training loads in the week following competition. These routine activities were of benefit as HRV and sAA returned to baseline values by the beginning of the next training week. The exact time course of these responses though remains to be clarified in future studies.

An interesting and noteworthy observation from the current study is the limited responses of HRV and sAA following the competition in week 9, in contrast to the decreased HRV and elevated sAA after the competition in week 6. Given the national swimming grand prix in week 9 was similar to the competition in week 6, with each athlete competing in up to 5 competitive races, the results suggest that each athlete coped more efficiently with the latter competition, resulting in negligible changes for HRV and sAA. However, the minimal fluctuations in HRV and sAA are most likely attributed to a number of other potential influences. Firstly, the two following competitions incorporated a taper training phase, reducing training load heading into the competitions in week 9 and 13, potentially alleviating the long-term impact of competition on HRV and sAA. Secondly, following the HRV and sAA responses after the week 6 competition, the coaching staff increased the recovery protocols and reduced the training load after the week 9 and 13 competitions, also likely mitigating any long-term depression in HRV and elevation of sAA following the week 9 and 13 competitions. These combined adjustments to the training and recovery practices most likely account for the lack of significant change in HRV and sAA following the two competition weekends.

While sAA typically returns to baseline levels at 30 to 60 minutes post exercise [22], the distinctive results of the current study showed sAA was elevated on the Monday morning after the three day grand prix swimming competition. It is unknown if this was the peak of these responses as measures were not taken immediately post each event or during the three day competition. These results would appear to indicate an accumulative effect of multiple exercise bouts, evident at least 24 hours after the last event, with sAA levels potentially peaking before the Monday morning recording. In a similar way, elevated levels of cortisol were reported up to 24 hours after an elite rugby league match [35]. While a single exercise bout may result in an acute peak in sAA [22] and cortisol [37], continuous or repetitive elite level competition with minimal recovery time between bouts may result in an extended rise in sAA levels. The elite athletes with a disability in the current study competed in as many as five races during the weekend (Friday to Sunday) and exhibited elevated sAA levels on the Monday morning, over 48 hours after the first of multiple competitive races. Further research may clarify the time-course of post-exercise sAA and/or the impact of prolonged sAA increases on performance.

During the periodised swimming program used in the current study, there were no significant weekly differences in either HRV or saliva immune function measures. In a similar way, Edmonds et al [7] observed comparable unchanged cardiac autonomic balance in elite Paralympic swimmers during a periodised swimming program in the lead up to the London 2012 Paralympic Games. Although there was no apparent change in cardiac autonomic activity, a recognised marker of training adaptation [38], each current athlete recorded personal best times during the competition period, suggesting the program inclusive of recovery protocols was well managed for the athletes involved. The lack of change in cardiac autonomic activity in the current study may be attributed to a number of potential factors. Firstly, it could be suggested that the absence of variation in HRV may be a result of the HRV indices used for analysis, however careful consideration was given when selecting the appropriate HRV measures for analysis. In particular, the natural logarithm of RMSSD, analysed in the current study, has been reported as the most reliable and practically applicable measure for elite athlete populations [4]. As such, the HRV indices analysed were well suited and sensitive for this research. Secondly, the potential for HRV saturation may also have been a factor that limited HRV change in the athletes. Considered somewhat normal in elite athlete populations [6], HRV saturation may limit any further HRV change with training [4]. Therefore, HRV may be limited as a marker of vagal modulation when vagal activity is already maximised, such as in athletes [39,40]. Lastly, the training stress for each athlete may have not been sufficient to induce a HRV and centralised training adaptation [39,40]. Despite the different training phases completed, training load remained relatively constant throughout the monitoring period which may have limited the potential for HRV change. However, as previously stated all athletes recorded personal best times during this study with each athletes’ performance benefiting positively from the implemented program. Furthermore, with a time difference of approximately 40 hours between the last training session and HRV recording, it is possible that each athlete had recovered sufficiently by the time of the HRV recording. With ANS recovery typically faster in elite athlete populations following exercise [41], this potentially explains the limited change in HRV and saliva measures over the monitoring period.

The most unique results of the current study were the relationships identified between sAA and both mean HR and SD1. These results demonstrated that an increase in HR corresponded with an increase in sAA and a decrease in SD1, reflective of increased sympathetic and/or decreased parasympathetic activity. While an early study suggested sAA was mediated primarily by the sympathetic nervous system [16], the current results suggested that sAA may also be influenced by parasympathetic activity. As such, sAA may be an easier and more sensitive tool than HRV to monitor ANS, and in particular parasympathetic, activity, a key indicator of cardiac health [24]. While HRV is a useful, non-invasive measure of ANS activity [4], it does require considerable data analysis, limiting the possibility for on-the-spot feedback. With recent updates in technology, point of care analysis is now available for sAA, with on-the-spot feedback now possible for coaches. Therefore, similar to HRV, sAA responses to training loads of varying intensities may provide beneficial information for coaches since ANS activity has been shown to be a reliable tool in monitoring training load and fitness [42].

## Study Limitations

It must be noted that the current results were based on a relatively small, but elite sample size (n = 8) of elite athletes with a disability. However numbers of this size are common in research using elite athlete populations [2,43] with statistical analysis adjusted accordingly. In the current study, data was analysed using magnitude-based inferences to account for individual variations in HRV and saliva variables and to best examine the practical significance of the data when investigating smaller populations. Others have examined HRV using magnitude-based inferences, identifying likely and very likely changes in HRV in comparable small populations [32,44].

## Conclusion

The first of its kind, this study has documented HRV and saliva (sIgA and sAA) responses to chronic training in elite athletes with a disability. The current study has identified that high level national competition influences both HRV (SD1) and saliva biomarkers of stress (sAA). It was also observed in the present study that regular periodised training did not appear to elicit a significant change in both HRV or saliva markers of immunity (sIgA) and stress (sAA). Lastly, the current study is the first to document a relationship between HRV and sAA. Further investigation is warranted in other athlete populations, including able-bodied athletes and elite athletes with varying disabilities to identify the most suitable HRV or saliva variables for training prescription and athlete monitoring. Enhancing the knowledge of this area will enable coaches and sports scientists to better monitor athletes, with the ultimate aim of improving athletic performance.
